# Platelet Activating Factor Enhances Synaptic Vesicle Exocytosis Via PKC, Elevated Intracellular Calcium, and Modulation of Synapsin 1 Dynamics and Phosphorylation

**DOI:** 10.3389/fncel.2015.00505

**Published:** 2016-01-08

**Authors:** Jennetta W. Hammond, Shao-Ming Lu, Harris A. Gelbard

**Affiliations:** Center for Neural Development and Disease, University of RochesterRochester, NY, USA

**Keywords:** platelet activating factor, PAF, presynaptic plasticity, synaptic vesicle pools, PKC, calcium, synapsin, readily releasable pool

## Abstract

Platelet activating factor (PAF) is an inflammatory phospholipid signaling molecule implicated in synaptic plasticity, learning and memory and neurotoxicity during neuroinflammation. However, little is known about the intracellular mechanisms mediating PAF’s physiological or pathological effects on synaptic facilitation. We show here that PAF receptors are localized at the synapse. Using fluorescent reporters of presynaptic activity we show that a non-hydrolysable analog of PAF (cPAF) enhances synaptic vesicle release from individual presynaptic boutons by increasing the size or release of the readily releasable pool and the exocytosis rate of the total recycling pool. cPAF also activates previously silent boutons resulting in vesicle release from a larger number of terminals. The underlying mechanism involves elevated calcium within presynaptic boutons and protein kinase C activation. Furthermore, cPAF increases synapsin I phosphorylation at sites 1 and 3, and increases dispersion of synapsin I from the presynaptic compartment during stimulation, freeing synaptic vesicles for subsequent release. These findings provide a conceptual framework for how PAF, regardless of its cellular origin, can modulate synapses during normal and pathologic synaptic activity.

## Introduction

Platelet activating factor (PAF) is an inflammatory phospholipid signaling molecule implicated in synaptic plasticity, learning and memory, and neurotoxicity (Clark et al., 1992; Wieraszko et al., 1993; Gelbard et al., 1994; Izquierdo et al., 1995; Teather et al., 1998; Xu et al., 2004). Because PAF can alter synaptic plasticity as well as stimulate the immune system (Zimmerman et al., 2002), PAF may fuel neuroinflammation and thus play multiple roles in neurodegenerative disease, regardless of the etiology. PAF levels are elevated in the CNS of patients or rodent models with multiple sclerosis, HIV associated neurocognitive disorders (HAND), seizure, trauma, neuropathic pain, and stroke; suggesting disturbed PAF production and metabolism in disorders with a neuroinflammatory component (Kumar et al., 1988; Lindsberg et al., 1990; Bazan et al., 1991; Gelbard et al., 1994; Callea et al., 1999; Akisu et al., 2003). Although most cell types including neurons can produce PAF (Maclennan et al., 1996; Aihara et al., 2000; Zimmerman et al., 2002), the cell type(s) generating pathological levels of PAF in the above conditions is/(are) unknown and may be different for each disorder. However, as stimulated immune cells (macrophages, neutrophils, microglia, etc.) generate large quantities of PAF by up-regulating expression and/or activity of the PAF synthesizing enzyme LPCAT2, they are likely central producers in neuroinflammatory disorders (Morimoto et al., 2010; Okubo et al., 2012; Morimoto et al., 2014).

Multiple studies have shown that PAF can exacerbate the inflammatory environment by stimulating inflammatory monocyte and neutrophil chemotaxis, activation, and production of TNF-α, Il-6, and reactive oxygen species (Czarnetzki and Benveniste, 1981; Del Sorbo et al., 2001; Kihara et al., 2005; Boetkjaer et al., 2007; Belanger et al., 2008; Forsman et al., 2013). In addition to the inflammatory action of PAF, high doses of PAF can be directly neurotoxic. PAF makes neurons vulnerable to NMDA receptor-dependent excitotoxic injury and can induce apoptosis (Gelbard et al., 1994; Xu et al., 2004; Bellizzi et al., 2005). Sub-lethal doses cause dendritic beading, loss of spines, and mitochondrial dysfunction (Perry et al., 1998, 2005; Parker et al., 2002; Bellizzi et al., 2005). Pharmacological or genetic inhibition of the PAF receptor (PAFR) in rodent models of neuroinflammatory disease successfully decreases neurological damage and inflammation (Kihara et al., 2005; Farooqui et al., 2006; Belayev et al., 2008; Eggert et al., 2009; Musto and Samii, 2011; Rodrigues et al., 2011; Okubo et al., 2012; Motoyama et al., 2013). These studies validate the PAF/PAFR associated signal cascade as viable targets for reducing inflammatory damage to the CNS and warrant further study into the mechanisms of how an overabundance in PAF signaling contributes to synaptic damage and the continuing harmful cycle of neuroinflammation.

PAF also plays a role in non-pathological neuronal signaling. In healthy brain, PAF enhances long-term potentiation (LTP) and improves performance in learning and memory tasks (Clark et al., 1992; Wieraszko et al., 1993; Izquierdo et al., 1995; Teather et al., 1998; Moriguchi et al., 2010). PAF is released from neurons by high frequency stimulation or NMDA receptor activation (Aihara et al., 2000). Both neurons and glia express the PAFR (Mori et al., 1996), which is a G protein coupled receptor that signals through Gαq but can also signal via Gαi (Shi et al., 1996; Ishii and Shimizu, 2000). The canonical Gαq pathway activates phospholipase C, which hydrolyzes phosphatidylinositol 4,5-bisphosphate (PIP2) to diacyl glycerol (DAG) and inositol trisphosphate (IP3). IP3 acts as a second messenger to mobilize intracellular calcium stores. DAG and calcium locally activate protein kinase C (PKC).

To the best of our knowledge, PAF’s mechanism of action at the synapse has been limited to a few reports focusing on its potentiation of LTP that have focused almost exclusively on postsynaptic readouts of activity (Bito et al., 1992; Clark et al., 1992; Wieraszko et al., 1993; Kato et al., 1994; Bellizzi et al., 2005; Moriguchi et al., 2010). Although it has long been thought that at least part of PAF’s LTP potentiation (physiological or pathological) is due to presynaptic mechanisms because PAF increases the frequency but not the amplitude of spontaneous miniature excitatory postsynaptic potentials (Clark et al., 1992; Kato et al., 1994), there has been little to no investigation into the downstream signaling pathways or molecular events within the presynaptic bouton mediating this up regulation of presynaptic activity. In order to investigate whether PAF directly influences neurotransmitter release and what intracellular mechanisms are involved, we used optical assays of exocytosis that directly measure presynaptic function. We show that PAF increases presynaptic vesicle exocytosis through PKC activation and elevated intracellular calcium within presynaptic boutons. PAF increases the size or release kinetics of the readily releasable pool (RRP) and total recycling pool of synaptic vesicles. We also report increased phosphorylation of synapsin I at sites 1 and 3 and greater dispersion of synapsin I from synaptic vesicles upon exposure to PAF. These findings provide mechanistic detail on how PAF specifically alters neurotransmitter release which has important implications both for normal physiology and pathological conditions involving neuroinflammation.

## Materials and Methods

### Cell Culture

Primary hippocampal cultures were prepared from embryonic day 18 Sprague–Dawley rats. Animal care and use were carried out in accordance with the recommendations of the Guide for the Care and Use of Laboratory Animals and protocols were approved by the University Committee on Animal Resources at the University of Rochester. Hippocampi were dissected out and dissociated in 0.05% trypsin (Life Technologies). Cells were plated at a density of 330/mm^2^ onto poly-D-lysine coated coverslips in neurobasal media supplemented with B27 with antioxidants, GlutaMAX (all reagents from Life Technologies), 5% FBS (Atlanta Biological), and 25 μM glutamic acid (Sigma). Cells were fed every 3–4 days with neurobasal media supplemented with B27 without antioxidants and GlutaMAX. At 7 days *in vitro* (DIV), cells were treated with 5-fluoro-deoxyuridine (fUDR) at 20 mg/mL and uridine at 50 mg/mL to limit glial growth. Cells were transfected at 14–15 DIV using a calcium phosphate transfection kit (Clontech). All experiments were performed at 18-21 DIV.

### Immunofluorescence

Cells were fixed with 4% paraformaldyhyde in PBS for 10 min and then treated with 100 mM glycine in PBS for 5 min. Cells were permeabilized with 0.1% triton-X (Promega) in PBS for 5 min. After blocking with 4% normal goat serum (Vector Laboratories) in PBS for 30 min, cells were incubated for 1.5 h using these primary antibodies: PAFR (Bioss bs-1478R), vGlut (EMD Millipore #5905), PSD95 (NeuroMAB clone K28/43 cat#75-028), vGAT (Synaptic Systems #131 004), and Gephrin (Synaptic Systems #147 021) in blocking buffer. Cells were washed three times with PBS then incubated with alexa-488, 568, and 657 conjugated secondary antibodies (Life Technologies) for 1 h. After washing cells three times in PBS, cells were mounted on glass slides using prolong gold with 4′,6-diamidino-2-phenylindole (DAPI; Life Technologies). Images were acquired using 0.2 μm *z*-steps on an Olympus BX-51 upright microscope with Quioptic Optigrid optical sectioning hardware using a 60x oil objective (NA 1.4) and a Hamamatsu ORCA-ER camera. Images were analyzed in Volocity (PerkinElmer Life and Analytical Sciences). Co-localization was determined using an automatic threshold according to Costes et al. (2004); and reported as Pearson’s co-localization coefficient. To quantify the percent of vGlut or PSD95 puncta touching PAFR puncta we used the find object function on flattened z stacks to select PAFR, vGlut, or PSD95 puncta using a local contrast adjustment with a 5 μm radius. The percent of vGlut or PSD95 puncta touching PAFR puncta was then calculated. For figures, digital image interpolation was used to increase resolution and enlarge the images in order to reduce pixelation.

### Live Cell Imaging and Field Stimulation

Neurons were imaged using an Olympus IX70 inverted microscope with a 60x 1.4NA objective or 40x 0.85NA objective with 1.5x Optivar. Images were acquired with a CCD camera (Q imaging Retiga Exi Fast). Coverslips were mounted in a custom made 75 μl volume, field stimulation chamber containing a modified Tyrode buffer consisting of: 124 mM NaCl; 5 mM KCl; 2 mM MgCl2; 2 mM CaCl2; 25 mM HEPES (pH 7.4); 30 mM glucose; and included 10 μM CNQX (Enzo Life Sciences) and 50 μM APV (Tocris) to block recurrent AMPA and NMDA receptor-mediated activity, respectively. Stimulus trains consisting of 1ms pulses at 30 mA were applied across parallel platinum electrodes spaced 8mm apart using a SIU-102 stimulator (Warner Instruments). Experiments were performed at room temperature. Somatic regions were excluded from all synapse measurements. For experiments with NH_4_Cl treatment, 50 mM NaCl of modified Tyrode buffer was replaced with 50mM NH_4_Cl (buffered to pH 7.4). When indicated, neurons were treated with 1 μM cPAF (a non-hydrolyzable form of PAF used to eliminate potential confounds from PAF catabolism due to endogenous acetylhydrolases; from Sigma or Enzo Life Sciences) or vehicle. cPAF was dissolved in modified Tyrode buffer for all experiments except those involving FM1-43 where it was first dissolved in ethanol then further diluted in modified Tyrode buffer. Final ethanol concentration for the FM1-43 experiments was 0.01%.

### Syn-pHluorin

Neurons were transfected with plasmids for farnesylated-Tdtomato (kind gift of Marc Halterman; University of Rochester) and pcDNA3-Syn-pHluorin 4x (a gift from Stephen Heinemann, Salk Institute for Biological Studies and Yonling Zhu, Northwestern University; Addgene plasmid 37005; Zhu et al., 2009). Neurons were stimulated two times with 100 pulses at 10 Hz separated by 3 or 5 min then treated with vehicle or cPAF for 2 or 20 min followed by two additional 100 pulse stimuli at 10 Hz. Images were taken every 2 s for 1 min intervals with the 100 pulse stimuli, beginning after six baseline images. Finally NH_4_Cl was added to raise internal vesicle pH, unmasking the total pHluorin fluorescence. In some experiments, cells were pretreated 25 min with 10 μM of a cell-permeable PKCα/β inhibitor peptide 20–28 (Myr-N-FARKGALRQ-NH2, referred to as PKCi; EMD Millipore) or 10 μM PAFR antagonist (BN 52021; Santa Cruz Biotechnology). Image stacks were analyzed in ImageJ and regions of interest (ROI) were drawn manually around NH_4_Cl responsive puncta. For each ROI, the average value of the first six baseline images before each stimulus was subtracted to calculate the Δ*F*. All fluorescence measurements from each bouton were normalized to the maximal NH_4_Cl response. An NH_4_Cl minimum response threshold was set at Δ*F* > 50 (arbitrary fluorescence units) to ensure there was enough dynamic range for measurement of Syn-pHluorin response to small stimuli. Additionally, boutons that showed peak fluorescence’s greater than 2.5X the baseline’s standard deviation during all four 100 pulse stimuli were included in the data set. Separately, silent boutons were defined as those where the response to the 100 pulse stimuli was smaller than 2.5X the baseline’s standard deviation. Synapses under this threshold were then manually inspected to confirm no discernable increase in pHluorin fluorescence above noise after stimulation. Percent change values were calculated as the mean peak Δ*F*/Δ*F*_NH4Cl_ values for the two stimuli after treatment minus the mean peak Δ*F*/Δ*F*_NH4Cl_ values for the two stimuli before treatment divided by the mean peak Δ*F*/Δ*F*_NH4Cl_ values for the two stimuli before treatment (Δ*F* Δ*F*/Δ*F*_NH4Cl (after treatment)_ – Δ*F*/Δ*F*_NH4Cl (before treatment)_)/Δ*F*/Δ*F*_NH4Cl (before treatment)_). Δ*F*/Δ*F*_NH4Cl_ traces were analyzed using Prism: Graph Pad and fit to a single exponential function.

To measure the total recycling pool using Syn-pHluorin, primary neuronal cells expressing Syn-pHluorin 4x were treated 20 min with 1 μM cPAF or vehicle followed by addition of 0.5 μM bafilomycin (EMD Millipore). Images were taken every 2 s with a 1000 pulse stimuli at 10 Hz given after 10 baseline images. Finally NH_4_Cl was added to reveal the total pHluorin fluorescence. Image stacks were analyzed in ImageJ and Prism as outlined above.

### Styryl Dye Recycling (FM1-43)

Presynaptic terminals were labeled with 10 μM FM1-43FX (Life Technologies) in modified Tyrode buffer using a stimulus train of 40 pulses at 20 Hz or 900 pulses at 10 Hz. FM dye was applied to cells 5 s before stimulation and was removed 30 s after the stimulus train ended. Neurons were washed with 500 μM Advasep-7 (Biotium) in modified Tyrode buffer for 30 s then washed by perfusion of modified Tyrode buffer for 3 min before imaging. Destaining was performed by stimulation with 900 pulses at 10 Hz. After one load and unload cycle, neurons were treated for 20 min with 1 μM cPAF or vehicle. FM dye was then loaded and unloaded two more times. Background correction was performed in ImageJ with the rolling ball algorithm (20 pixel radius), which is designed to correct for unevenly illuminated background. Image stacks were analyzed in Volocity. A maximally loaded image (after 900 pulse load) was used to identify FM positive synaptic puncta using the Find Spot function. This function identifies local intensity maxima above a set threshold within a radius of 0.75 μm. A circular ROI, 1 μm in diameter, was placed around each identified spot to track fluorescent changes for that puncta over time. Only puncta whose fluorescence after the 900 pulse load decreased by 50% or more after an unload cycle were included in the data set.

### Calcium Measurements

Cells transfected with plasmids for Synaptophysin-GCaMP2 (CMV::ratSyGCaMP2 was a gift from Leon Lagnado, University of Sussex; Addgene plasmid # 26124; Dreosti et al., 2009) and farnesylated Tdtomato were mounted in a custom-designed chamber onto the microscope. After establishing baseline fluorescent measurements for 1–1.5 min (imaging every 10 s), 2 μM cPAF in was delivered locally onto the field of view using a 4-channel drug delivery system (ALA Scientific Instruments) and cells were imaged for another 5 min. For measurements of calcium influx evoked by a stimulus of 10 pulses at 10 Hz, images were obtained at a frequency of 1 Hz. Image stacks were analyzed using ImageJ software. ROI were manually drawn around Syn-GCaMP2 puncta and average fluorescence was measured at each time point using the time series analyzer plugin (created by J. Balaji). A local background measurement was taken adjacent to each puncta and then subtracted from the selected puncta’s fluorescent value. In many samples there were small diminutions in fluorescence over time during baseline measurements. To correct for this, we fit a straight line to the baseline measurements as a function of time and used the slope to correct all F measurements before and after vehicle or PAF treatment. The Δ*F*/*F*_0_ of each puncta was calculated as the difference between *F* at each time point (*F*_t_) and the baseline *F* (*F*_0_) (average *F* from 10–15 frames before treatment) divided by the average baseline *F* (*F*_0_).

### Synapsin I Dispersion

Once a field of view was identified with synapses expressing Synapsin I-GFP (gift of Timothy Ryan, Weill Cornell Medical College) and farnesylated Tdtomato, cells were treated with vehicle or 1 μM cPAF and images were obtained every 5 s. After 2 min (time 0), a 900 pulse stimulus train at 10 Hz was given. Background correction was done using ImageJ with the rolling ball algorithm (20 pixel radius). ROI were manually drawn around Synapsin I-GFP-expressing puncta. The Δ*F*/*F*_0_ of each Synapsin I-GFP puncta was calculated as the difference between *F* at each time point (*F*_t_) and F at time 0 (*F*_0_), then normalized to F_0_.

### Western Blots

Cells were treated for 0, 2, or 20 min with 1 μM cPAF dissolved in modified Tyrode buffer and diluted in cell media. Some cultures were also treated for a total of 30 min with 10 μM CNQX and 50 μM APV. Cells were then rinsed in ice cold PBS before being scraped into RIPA lysis buffer containing protease and phosphatase inhibitor cocktails (Sigma, Cat. # P8340 and EMD Millipore: Calbiochem, SetV, respectively). The cell lysate was kept on ice with periodic vortexing for 30 min then centrifuged at 13,000 rpm for 10 min. The supernatant was mixed with loading dye, heated at 95°C for 5 min, ran on a 4–15% SDS-PAGE gel, and transferred to nitrocellulose. Membranes were blocked with 5% milk in TBSt for 30 min and probed with primary antibodies (phospho-synapsin Ser9 (Cell Signaling Technologies #2311), phospho-synapsin I Ser603 (Rockland 612-401-C95), and GAPDH (EMD Millipore: Calbiochem CB1001) in 3% BSA in TBSt at 4° overnight. Membranes were washed three times in TBSt and then incubated with HRP secondary antibodies for 1 h in 5% milk in TBSt. After washing we applied ECL substrate (Pierce) and exposed and developed membranes on film.

## Results

### The PAFR is Located at Synapses

**FIGURE 1:**
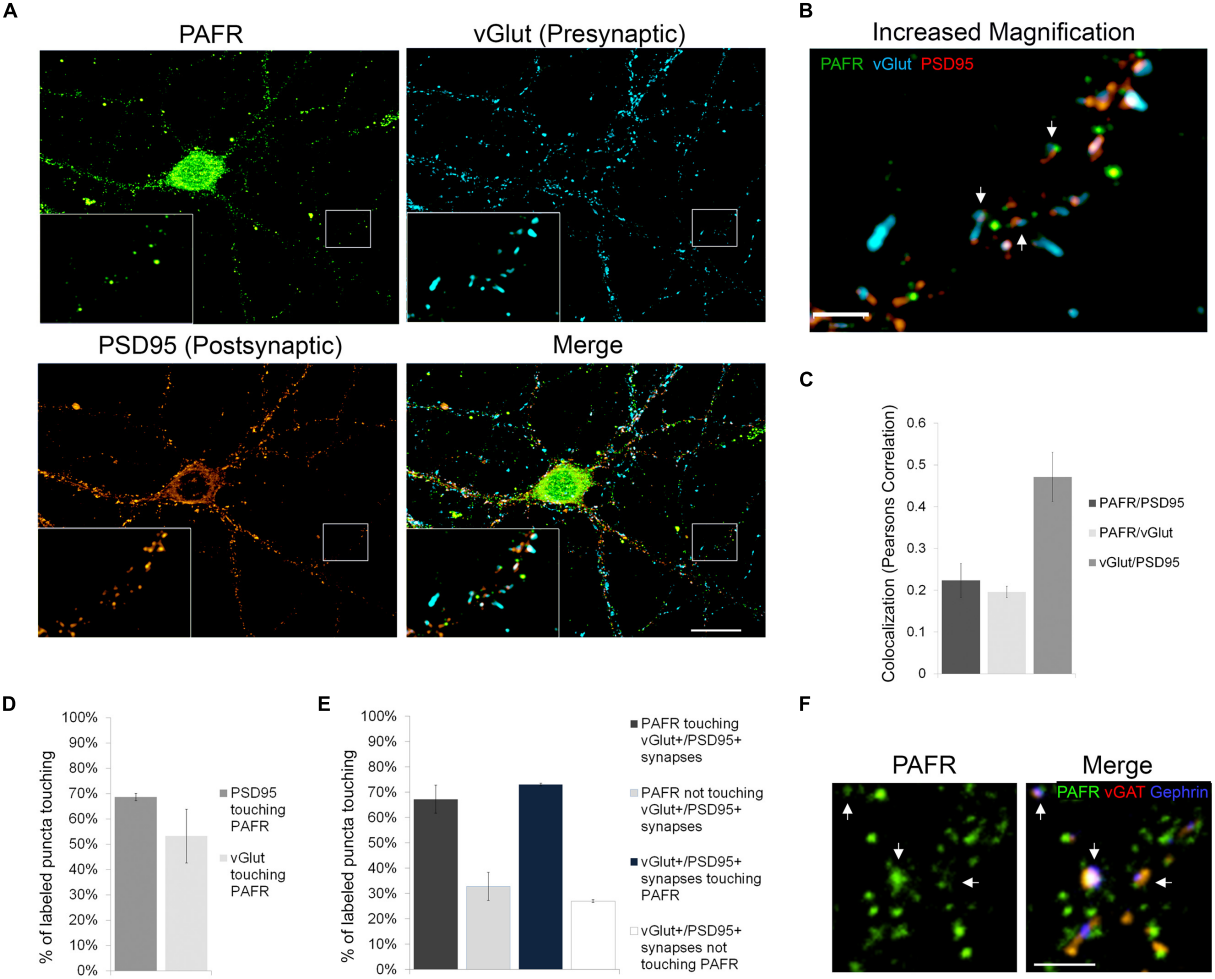
**The PAFR co-localizes to synapses. (A,B)** Immunofluorescence of hippocampal neuronal cultures 21 days *in vivo* (DIV). PAFR (green); presynaptic marker vGlut (blue); postsynaptic marker PSD95 (red). **(A)** Boxed region is shown with 3× magnification in left corner and further magnified in **(B)**. Scale bars **A** = 20 μm, **B** = 3 μm. Arrows highlight PAFR puncta touching vGlut+/PSD95+ synapses. **(C)** Colocalization of PAFR, PSD95, and vGlut calculated as Pearsons correlation coefficient. **(D)** Quantification of the percent of PSD95 or vGlut puncta touching PAFR puncta. **(E)** Quantification of the percent of PAFR puncta that contact vGut+/PSD95+ synapses and the percent of vGlut+/PSD95+ synapses that contact PAFR puncta. Error bars represent ±SEM. **(F)** Immunofluorescence of hippocampal neuronal cultures. PAFR (green); inhibitory presynaptic marker vGAT (red); inhibitory postsynaptic marker Gephrin (blue). Scale bar = 3μm. Arrows highlight PAFR puncta touching vGAT+/Gephrin+ synapses.

In order to better understand the functions of PAF signaling in neurons, we first investigated the subcellular localization of the PAFR. We immunolabeled primary hippocampal neurons with antibodies to the PAFR and presynaptic (vGlut) and postsynaptic (PSD95) markers (**Figures 1A,B**). We observed that PAFR puncta were located throughout the cell body and neuronal processes and were also found in glia (**Figure 1A**; Supplementary Figure S1A). A second PAFR antibody targeting a separate epitope showed a similar staining pattern in neurons (Supplementary Figures S1B–D). We also noted strong association of PAFR puncta with glutamatergic synapses. 53% of vGlut puncta contacted PAFR puncta (Pearson’s correlation coefficient for co-localization: 0.20 ± .01) and 69% of PSD95 puncta contacted PAFR puncta (Pearson’s correlation coefficient for co-localization: 0.22 ± .04; **Figures 1C,D**). When evaluating synapses that are positive for both vGlut and PSD95, 73% of synapses contacted PAFR puncta and 67% of PAFR puncta contacted vGlut+/PSD95+ synapses (**Figure 1E**). Some of the remaining PAFR puncta co-localized with inhibitory synapses (vGAT+/gephrin+) (**Figure 1F**) and some were likely non-synaptic.

### cPAF Enhances Presynaptic Vesicle Exocytosis

**FIGURE 2:**
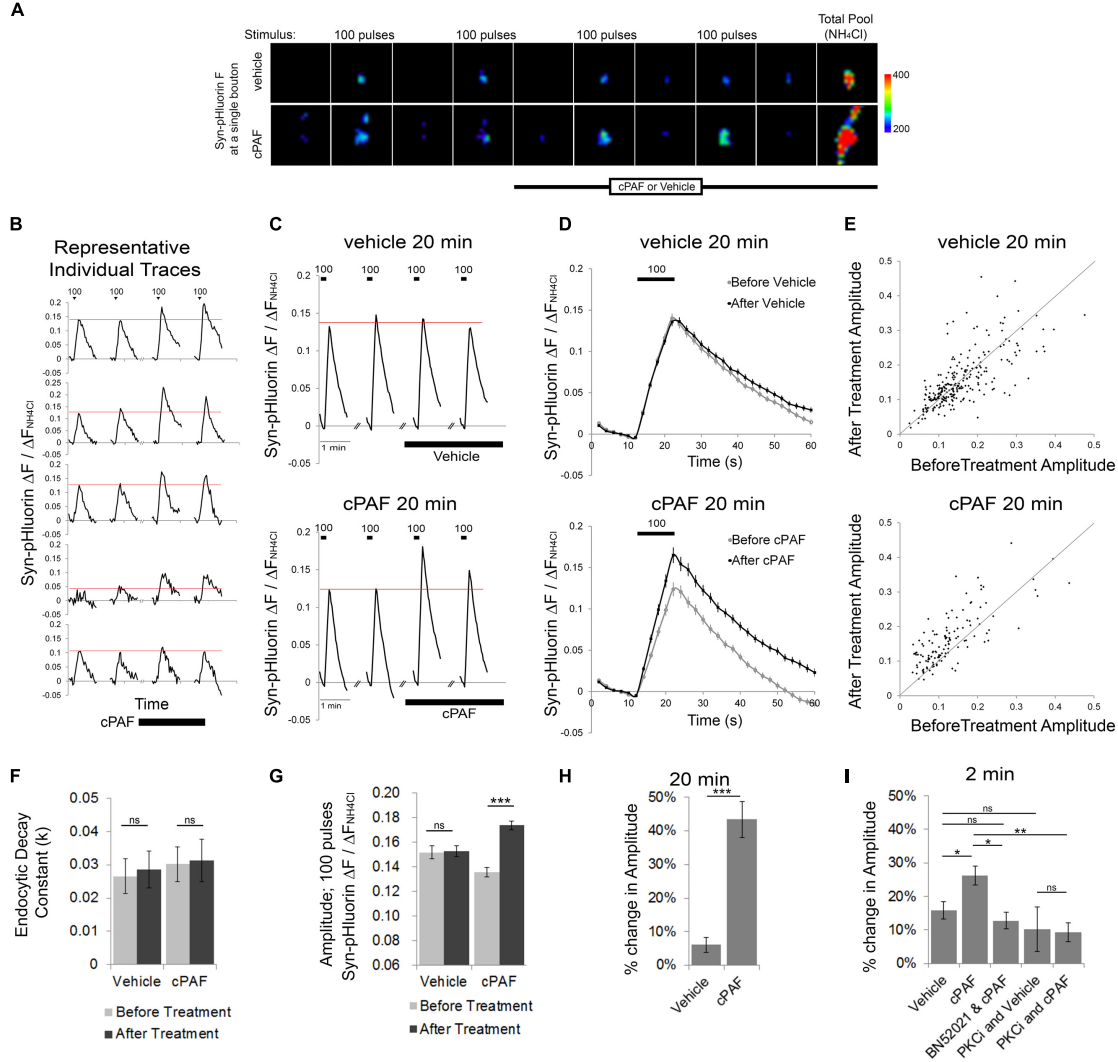
**cPAF enhances presynaptic vesicle exocytosis. (A–D)** The Syn-pHluorin fluorescence (F) at single boutons (from primary hippocampal cultures transfected with Syn-pHluorin) increases after each 100 pulse stimuli at 10 Hz as a portion of labeled vesicles are exocytosed and then returns to baseline as synaptic vesicles are internalized. Treatment with NH_4_Cl raises the pH of all internal vesicle and results in maximal pHluorin fluorescence representative of the total vesicle pool. All Syn-pHluorin measurements are normalized to the Δ*F* with NH_4_Cl. **(A)** Representative fluorescence images of single boutons treated with 1 μM cPAF or vehicle. Images are 4.3 μm × 4.3 μm. **(B)** Examples of individual traces (Δ*F*/Δ*F*_NH4Cl_) from representative boutons treated with 1 μM cPAF. **(C)** Traces show the average Syn-pHluorin Δ*F*/Δ*F*_NH4Cl_ over time for all boutons treated with 1 μM cPAF or vehicle. Vehicle, 20 min: 263 boutons from seven coverslips; cPAF, 20 min: 120 boutons from five coverslips. **(D)** Average Δ*F*/Δ*F*_NH4Cl_ response to the two before treatment 100 pulse stimuli shown overlapping the average response to the two 100 pulse stimuli given after 1 μM cPAF or vehicle treatment. **(E)** Scatter plot of the average peak amplitude of Δ*F*/Δ*F*_NH4Cl_ induced by 100 pulse stimuli before and after 20 min treatment with vehicle or 1 μM cPAF. **(F)** Non-linear regression of decay part of Δ*F*/Δ*F*_NH4Cl_ trace (in **D**) was used to quantitate the endocytic decay constant (*k*) followed by testing the null hypothesis of one rate for all data sets (*p* = 0.807). **(G)** Average peak amplitude of Δ*F*/Δ*F*_NH4Cl_ induced by 100 pulse stimuli before and after 20 min treatment with vehicle or 1 μM cPAF. Statistical analysis was performed using two-way ANOVA with repeated measures followed by Sidak’s multiple comparison tests. **(H)** Quantification of the % change in peak amplitude due to 20 min treatment of vehicle or 1 μM cPAF. Statistical analysis used paired Student’s *t*-test. **(I)** Quantification of the % change in peak amplitude due to 2 min treatment of vehicle or 1 μM cPAF. Some samples were also pretreated 30 min with BN52021 (a PAFR inhibitor) or PKCi (a PKC inhibitor). Statistical analysis used one-way ANOVA with Sidak’s multiple comparison tests. For all graphs statistical significance is indicated by the following markings: ^∗^*p* < 0.1; ^∗∗^*p* < 0.01; ^∗∗∗^*p* < 0.001; ns = not significant. Error bars are ±SEM.

To determine whether PAF exposure alters presynaptic vesicle release and organization of vesicles within different synaptic vesicle pools, we used optical monitoring of the presynaptic vesicle protein synaptophysin labeled on its luminal side with four tandem pH sensitive GFPs (Syn-pHluorin). Syn-pHluorin fluorescence is quenched by the low pH inside synaptic vesicles but fluoresces strongly when it encounters neutral pH at the cell surface during exocytosis. Endocytosis of Syn-pHluorin and reacidification of the vesicle returns Syn-pHluorin to a quenched state. We measured the changes in Syn-pHluorin fluorescence at individual synaptic boutons evoked by 100 pulse stimuli at 10 Hz before and after cPAF or vehicle treatment (**Figure 2**). All fluorescence values were normalized to the change in fluorescence resulting from exposure to NH_4_Cl which rapidly raises the pH of internal vesicles revealing the total pool of labeled vesicles in order to correct for possible variation in expression levels. A 20 minute cPAF treatment caused a significant increase in the peak amplitude of Syn-pHluorin fluorescence in response to 100 pulse stimuli (**Figures 2B,E,G**); Average peak amplitude before cPAF 0.136 ± 0.008; after cPAF 0.174 ± 0.009; Average peak amplitude before vehicle 0.152 ± 0.005; after vehicle 0.153 ± 0.005). cPAF did not affect the fluorescence decay rate revealing that endocytosis was not significantly changed by cPAF treatment (**Figure 2F**). Thus the rise in peak amplitude due to cPAF treatment (a 43.4 ± 5.4 % increase; **Figure 2H**) is likely due primarily to enhanced exocytosis. Boutons treated with cPAF for a much shorter time period, 2 min, showed a smaller, yet significant increase in peak amplitude (26.2 ± 2.8%) as opposed to vehicle treated boutons. (16.0 ± 2.5%) (**Figure 2I**).

### cPAF Increases Vesicle Release by Signaling Through the PAFR and PKC

We next tested whether cPAF’s enhanced vesicle release was receptor specific and whether it involves PKC by using pharmacological inhibitors. Pretreatment with a PAFR antagonist (BN52021) prevented the cPAF induced increase in Syn-pHluorin amplitude (**Figure 2I**). The cPAF induced potentiation was also prevented in the presence of a peptide PKC inhibitor (PKCi; **Figure 2I**) indicating that PAFR signaling enhances synaptic vesicle exocytosis by activating PKC downstream of the PAFR.

### cPAF Activates Presynaptically Silent Synapses

**FIGURE 3:**
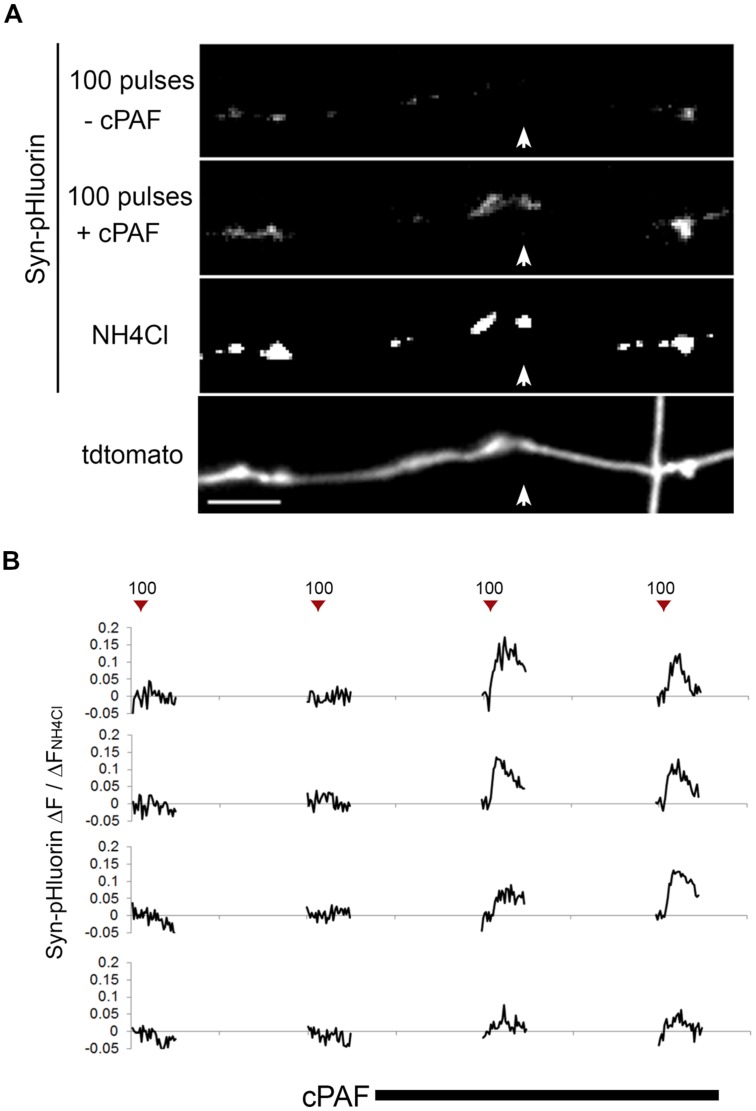
**cPAF activates silent presynaptic boutons. (A)** Images show average peak Δ*F* of Syn-pHluorin upon stimulation of 100 pulses at 10 Hz or NH_4_Cl treatment. A small percentage of synapses show no measurable exocytosis following stimulation, although acidification of all vesicles by NH_4_Cl shows Syn-pHluorin is present in these boutons (example highlighted by white arrow). Note that after cPAF treatment (20 min) all boutons imaged show potentiation due to cPAF and the previously silent boutons are now robustly active. Scale bar 5 μm. **(B)** Representative Syn-pHluorin Δ*F*/Δ*F*_NH4Cl_ traces of silent boutons that were activated by 1 μM cPAF (20 min). The first trace corresponds to the bouton in **(A)** highlighted by the white arrow.

Because cPAF can potentiate exocytosis from active boutons, we next looked at whether cPAF could activate presynaptically silent synapses (i.e., those releasing no vesicles after an action potential). In our Syn-pHluorin experiments about 4% percent of synapses gave no measurable response to the 100-pulse stimuli despite the fact that NH_4_Cl treatment revealed the presence of a significant cluster of Syn-pHluorin (**Figure 3A**). cPAF treatment robustly activated a large percent of these silent synapses (60% with 2 min vs. 75% with 20 min cPAF treatment) (**Figure 3B**). Thus cPAF PAF treatment enhances presynaptic activity by both increasing the number of vesicles released from individual terminals and by increasing the number of active boutons.

### cPAF Enhances Presynaptic Activity by Increasing the Size or Release of the RRP

As cPAF enhances exocytosis induced by a 100-pulse stimuli (10 s at 10 Hz), we were interested in whether cPAF may influence the distribution of vesicles to different synaptic vesicle pools within presynaptic boutons. Synaptic vesicles within a presynaptic terminal can be subdivided into three functional pools. The RRP consisting of vesicles that are docked and primed at the active zone; the recycling pool of vesicles that replenish the RRP and are released upon continued stimulation; and the resting pool that are resistant to release. The RRP and the recycling pool together make up the total recycling pool. The size of the RRP and recycling pool or the efficiency in which the RRP refills from the recycling pool plays an important role in presynaptic strength (Alabi and Tsien, 2012).

**FIGURE 4:**
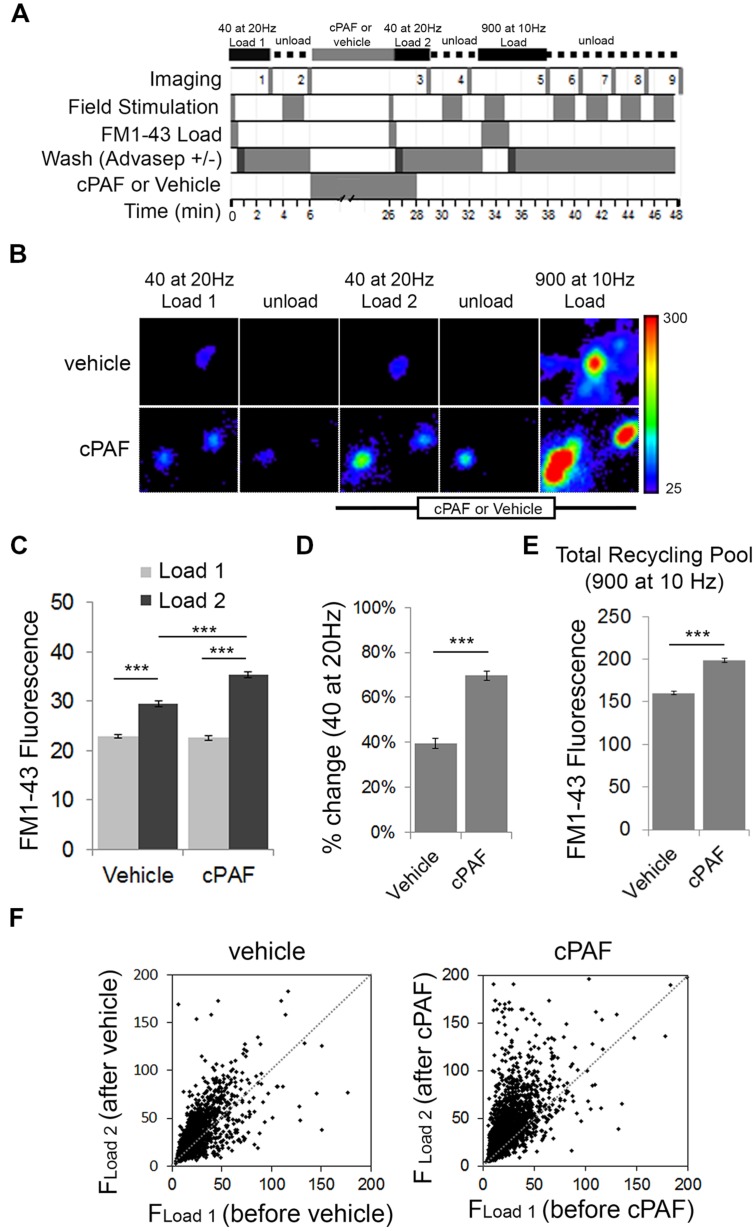
**cPAF enhances the size or release probability of the readily releasable pool (RRP) and the size of the total recycling pool as determined by FM1-43 dye uptake. (A)** Schematic of FM1-43 dye loading protocol. Cells were stimulated with 40 pulses (2 s at 20 Hz) to load the RRP (Load 1 = before treatment and Load 2 = after treatment). Cells were stimulated with 900 pulses (90 s at 10 Hz) to load the recycling pool or to unload FM dye. **(B)** Representative boutons from vehicle and cPAF treated samples. Scale of images = 4 μm × 4.6 μm. **(C)** Quantification of the average FM1-43 Fluorescence (±SEM) obtained for Load 1 and Load 2 of the RRP from boutons treated 20 min with vehicle or 1 μM cPAF (vehicle *n* = 2098 boutons from three coverslips; cPAF *n* = 3030 boutons from four coverslips). Statistical analysis was performed using two-way ANOVA followed by Sidak’s multiple comparison tests. **(D)** Quantification of the average % change [(*F*_Load2_–*F*_Load1_)/*F*_Load1_) in FM1-43 fluorescence due to vehicle or cPAF treatment. Statistical analysis was performed using Student’s *t*-test. **(E)** Quantification of the total recycling pool as the average FM1-43 Fluorescence (±SEM) in boutons after a 900 pulse at 10 Hz stimulation for neuronal cultures treated 20 min with vehicle or 1 μM cPAF. **(F)** Scatter plot of FM1-43 fluorescence before (*F*_Load1_) and after (*F*_Load2_) cPAF or vehicle treatment. For all graphs statistical significance is indicated by the following marking: ^∗∗∗^*p* < 0.001. Error bars ±SEM.

To test whether cPAF alters the size of the RRP and the total recycling pool we switched experimental methods to use field stimulation to load FM1-43 dye into presynaptic boutons. Fluorescent FM styryl dyes bind reversibly to cellular membranes and can be loaded and unloaded from synaptic vesicles upon stimulated vesicle cycling. After applying FM1-43 to neuronal cultures, a 40 pulse stimulus train at 20 Hz (a stimulation protocol often used to estimate the size of the RRP; Li et al., 2005) was given to stimulate synaptic vesicle exocytosis with compensatory endocytosis. After extensive washing, the fluorescence intensity from dye trapped within endocytosed synaptic vesicles provided an estimate of baseline RRP size (load 1). After unloading the dye and treating the neurons for 20 min with 1 μM cPAF or vehicle, the RRP of the same population of boutons was reloaded with FM1-43 (load 2). Following unloading, a final maximal load and unload cycle, each using a 900 pulse stimulus train was used to confirm the location of active boutons and measure the size of the total recycling pool (**Figure 4A**). Exposure to cPAF resulted in a significantly larger % increase in FM fluorescence during load 2 of the RRP compared to load 1 than exposure to vehicle (cPAF: 69.7 ± 2.1%; vehicle: 40 ± 2.3%; *p*<0.0001; **Figures 4B–D**). A small increase is expected as background fluorescence increases upon each exposure to FM dye. The distribution of FM fluorescence in individual boutons from load 1 to load 2 clearly shows a strong increase in FM loading after cPAF treatment, with boutons that have a low loading capacity showing the largest increase in relative fluorescence (**Figure 4F**). These data demonstrate that cPAF increases the quantity of synaptic vesicles released following the 40 pulses stimuli which is consistent with an increase in the size of the RRP or an increase in vesicles’ probability of release. cPAF also resulted in an increase in the size of the total recycling pool (**Figure 4E**).

### cPAF Increases the Rate of Exocytosis of the Total Recycling Pool

**FIGURE 5:**
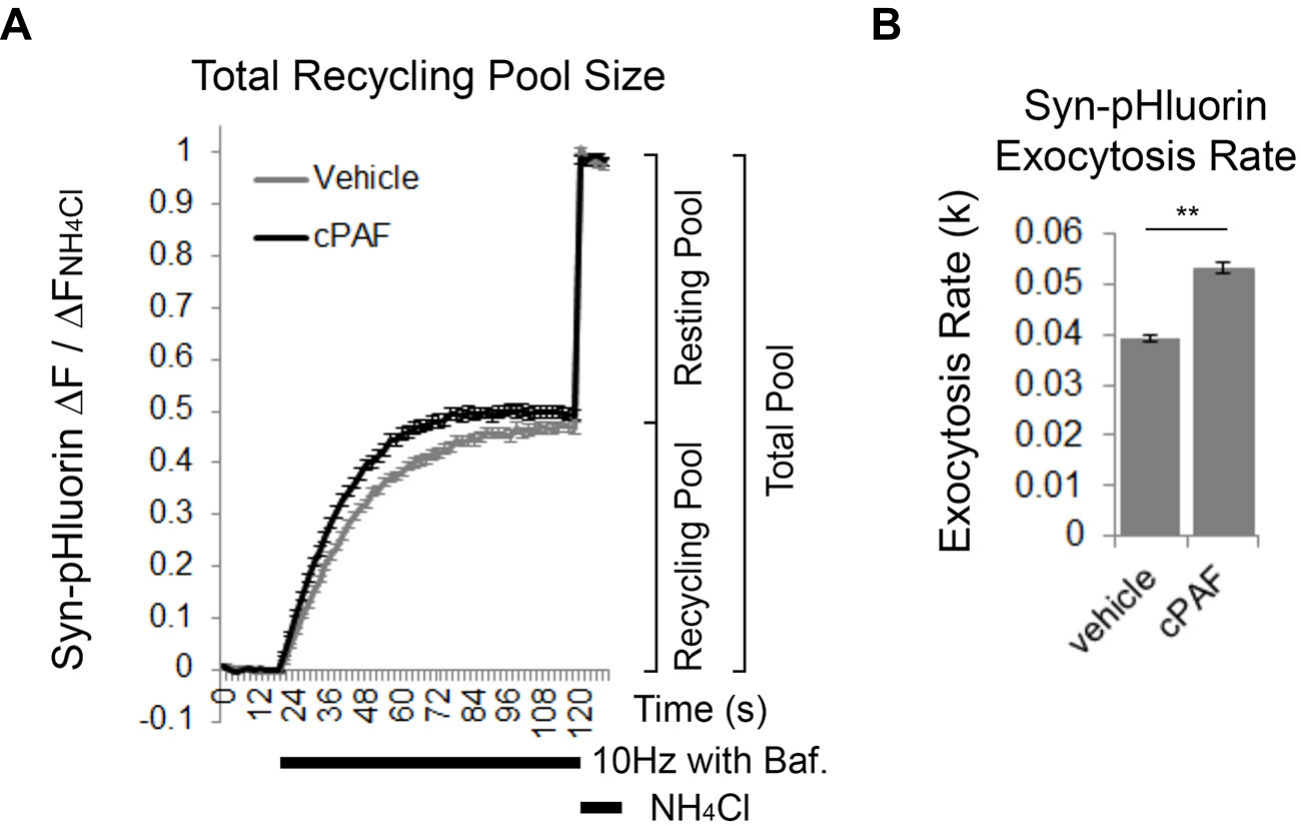
**cPAF increases the exocytosis rate of the total recycling pool. (A,B)** Cells, transfected with Syn-pHluorin, were stimulated with 1000 pulses at 10 Hz in the presence of bafilomycin (a proton pump inhibitor) to induce the release of all synaptic vesicles in the total recycling pool while preventing the reacidification of the vesicles after endocytosis that usually quenches pHluorin fluorescence. Following the stimulation, cells were treated with NH_4_Cl to measure the total pool of synaptic vesicles. **(A)** Average Syn-pHluorin Δ*F*/Δ*F*_NH4Cl_ traces from cells pretreated for 20 min with vehicle or 1 μM cPAF. (Plateau values at end of 1000 pulse stimulus = total recycling pool size: Vehicle 0.47 ± 0.01 (*n* = 534 synapses from seven coverslips; cPAF 0.50 ± 0.01 (*n* = 558 synapses from seven coverslips). Student’s *t*-test *p* = 0.09). **(B)** Syn-pHluorin Δ*F*/Δ*F*_NH4Cl_ traces were fit with a single exponential function by non-linear regression to determine the exocytosis rate constant (*k*) for the total recycling pool followed by testing the null hypothesis of one rate for all data. ^∗∗^*p* < 0.001 Error bars ±SEM.

We next used Syn-pHluorin as a second method to measure the size of the total recycling pool. Normally the Syn-pHluorin fluorescence signal during extended stimulation is a balance of exocytosis, endocytosis, and reacidifcation of the newly internalized vesicles. However, extended stimulation in the presence of bafilomycin, a proton pump inhibitor that prevents reacidification, causes the Syn-pHluorin signal to plateau because all vesicles that can undergo exocytosis have been released at least once and remain at neutral pH trapping pHluorin in its fluorescent state even though endocytosis and vesicle cycling is still continuing. The plateau fluorescence represents the size of total recycling pool. Following the stimulus, exposure of the boutons to NH_4_Cl neutralizes all internal vesicles and reveals the presence of any Syn-pHluorin containing vesicles in the resting pool that did not get released and thus remained in an acidic state during the stimulus. Pretreatment of boutons to cPAF resulted in a slight increase in the size of the total recycling pool compared to vehicle treatment (**Figure 5A**; cPAF 0.50 ± 0.01; vehicle 0.47 ± 0.01; *p* = 0.090), in agreement with the FM1-43 experiment (**Figure 4E**). However, cPAF more significantly increased the kinetics of exocytosis (**Figures 5A,B**). The rate of exocytosis of the recycling pool was significantly faster for cPAF treated boutons than vehicle treated (cPAF *k* = 0.039 ± 0.001; vehicle *k* = 0.053 ± 0.001; *p* < 0.001). Thus cPAF’s enhancement of exocytosis is possibly controlled at multiple levels including increased recycling pool and RRP size and enhanced mobilization between pools.

### cPAF Raises Intracellular Calcium Levels Within Presynaptic Boutons

**FIGURE 6:**
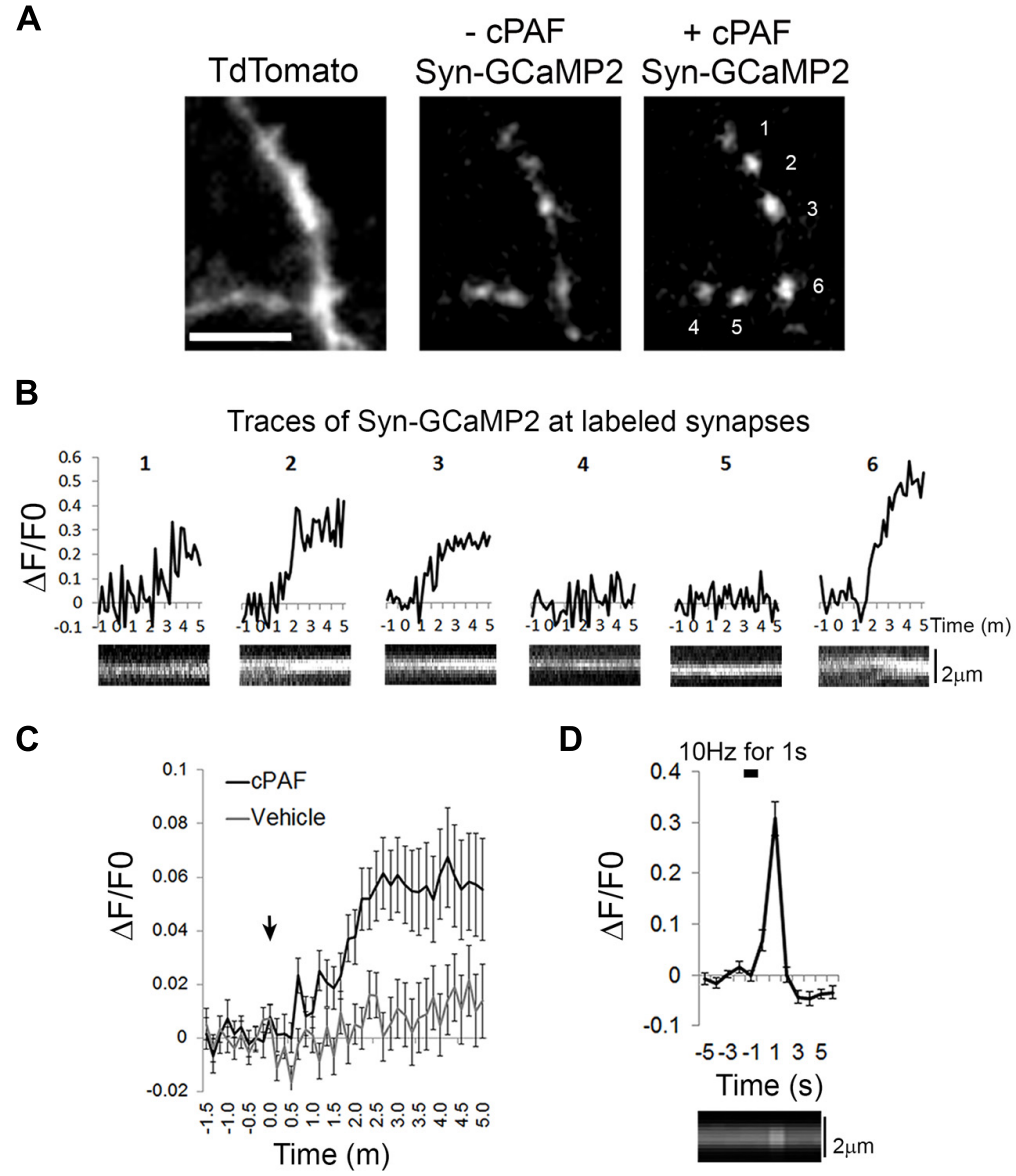
**cPAF raises calcium levels in presynaptic boutons. (A)** Hippocampal neurons expressing Tdtomato (to view axons) and the calcium indicator Syn-GCaMP2 before and after 5 min with 2 μM cPAF. Scale is 4 μm. **(B)** Traces showing Syn-GCaMp2 % change in fluorescence (Δ*F*/*F*_0_) over time from boutons numbered in **(A)** that were treated with cPAF at time 0. Under each trace is a kymograph showing the Syn-GCaMP2 fluorescence from a line scan through the same bouton. **(C)** Quantification of the average Syn-GCaMP2 % change (Δ*F*/*F*_0_) of fluorescence over time from all boutons treated with cPAF or vehicle. (cPAF: 71 boutons from five coverslips; vehicle: 59 boutons from three coverslips; Student’s *t*-test at 3 min *p* = 0.0010; Error bars are ±SEM. **(D)** Quantification of the average % change in fluorescence (Δ*F*/*F*_0_) over time evoked by a 10 Hz for 1 s field stimulus. (27 boutons from two coverslips; Error bars are ±SEM) Kymograph under graph shows the Syn-GCaMP2 fluorescence from a line scan through a representative bouton. Error bars are ±SEM.

As cPAF, signaling through the PAFR, has been shown to raise intracellular calcium levels in multiple cell types through intracellular stores (Ishii and Shimizu, 2000), we investigated whether cPAF specifically raises calcium levels in presynaptic boutons. We used the genetically encoded calcium indicator GCaMP2 attached to the cytoplasmic domain of synaptophysin (Syn-GCaMP2) to measure relative calcium levels within presynaptic boutons (Dreosti et al., 2009). cPAF treatment resulted in a small but significant sustained increase in intra-bouton calcium within 2–5 min (at 3 min cPAF: Δ*F*/*F*_0_ = 0.06 ± 0.01; vehicle Δ*F*/*F*_0_ = 0.01 ± 0.01; Student’s *t*-test p<0.001; **Figures 6A–C**). The response was heterogeneous with some boutons showing little to no change and others showing a much larger response (see example traces **Figure 6B**). As a control comparison, calcium entry due to a 10 pulse stimuli (1 s at 10 hz) in a separate set of boutons show calcium levels increase significantly higher than the average response to cPAF (Δ*F*/*F*_0_ = 0.30 ± 0.03; **Figure 6D**) but this calcium spike occurs much more quickly and is much briefer in duration. These results suggest that cPAF’s increase in presynaptic vesicle release involves a small, but sustained elevation of calcium within presynaptic boutons. As multiple forms of short-term presynaptic plasticity rely upon a buildup of residual calcium in boutons after a burst of action potentials (de Jong and Fioravante, 2014), this small intracellular rise in calcium could act similarly to potentiate vesicle release.

### cPAF Increases Synapsin I Dispersion and Phosphorylation

**FIGURE 7:**
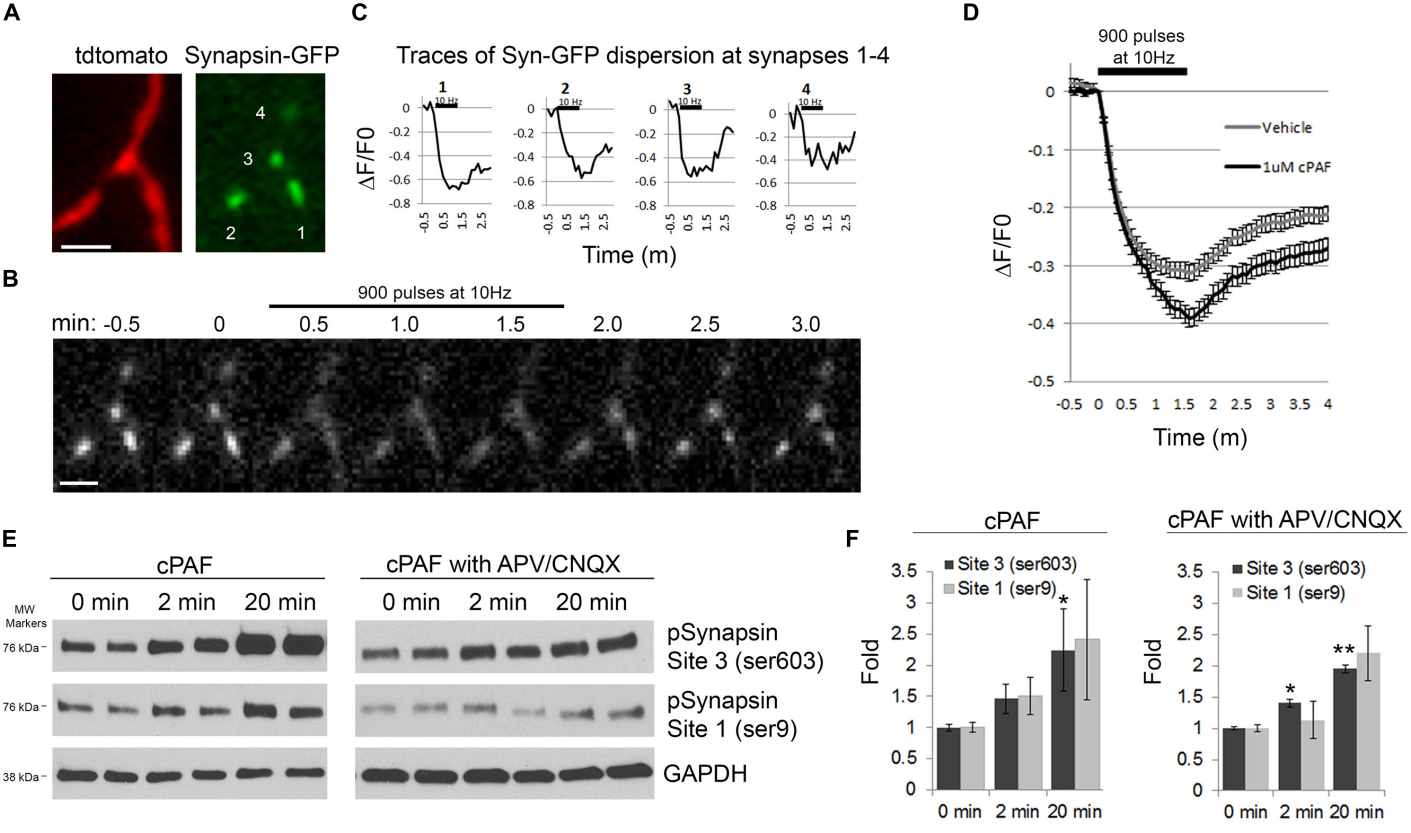
**cPAF increases synapsin I phosphorylation and synapsin I dispersion from presynaptic boutons during stimulation. (A–D)** Hippocampal neurons expressing synapsin I-GFP and Tdtomato were treated with 1 μM cPAF for 2 min then stimulated with 900 pulses at 10 Hz. **(A)** Synapsin I-GFP (Syn-GFP) is clustered at presynaptic boutons while tdtomato fills the entire axon. Scale bar = 2 μm. **(B)** Syn-GFP fluorescence at synapses decreases upon stimulation then reclusters within the bouton. Scale bar = 2 μm. **(C)** Timelapse measurements of the change in Syn-GFP fluorescence intensity obtained from the boutons numbered in **(A)** normalized to the fluorescence at time 0. **(D)** Average Δ*F*/*F*_0_ in syn-GFP fluorescence from all boutons treated with 1 μM cPAF or vehicle. Error bars ±SEM (cPAF: 141 boutons from three coverslips; vehicle: 240 boutons from four coverslips;). Non-linear regression of decay part of synapsin I dispersion curve followed by testing the null hypothesis of one curve for all data sets has *p* < 0.0001. Statistical analysis comparing cPAF to vehicle treated boutons at the end of stimulation (1.5 min) was done using the Student’s *t*-test *p* = 0.0005. **(E)** Representative western blots showing phosphorylated synapsin I (pSynapsin) at Site 3 (Ser603) and Site 1 (Ser9) and GAPDH immunoreactivity from cell lysates obtained from primary hippocampal cultures that were treated 0, 2, or 20 min with 1 μM cPAF. Blot on right was additionally treated for a total of 30 min with 50 μM APV and 10 μM CNQX to block glutamatergic neurotransmission. **(F)** pSynapsin/GAPDH ratios calculated by densitometric scanning of the blots. Data are means ± SEM with *n* = 4 (two independent experiments each performed in duplicate). Statistical analysis: one-way ANOVA followed by Dunnett’s multiple comparison test to 0 min control. ANOVA: Site 3, *p* = 0.098; Site 3 with APV/CNQX, *p* = 0.024; Site 1, *p* = 0.211; Site 1 with APV/CNQX, *p* = 0.126. Dunnett’s multiplicity adjusted *p*-values (compared to 0 min control): ^∗^*p* < 0.1 ^∗∗^*p* < 0.05.

Next we wanted to look at potential downstream mediators of cPAF’s enhancement of presynaptic activity. Synapsins are a good candidate as synapsins limit the mobility of the resting pool of synaptic vesicles and thus may be a major player in determining the size or release kinetics of the total recycling pool. Additionally, synapsin activity is controlled by phosphorylation/dephosphorylation, often in a calcium dependent manner (Chi et al., 2001, 2003; Cesca et al., 2010; Kim and Ryan, 2010; Bykhovskaia, 2011; Orenbuch et al., 2012; Verstegen et al., 2014). During extensive stimulation, synapsin I dissociates from synaptic vesicles and disperses from boutons freeing vesicles for release (Chi et al., 2001, 2003). Thus, we asked if cPAF’s enhancement of presynaptic vesicle release was associated with increased synapsin I dispersion. Presynaptic boutons from neurons transfected with GFP-tagged synapsin I and Tdtomato were stimulated with 900 pulses at 10Hz. Upon stimulation, the relative abundance of synapsin I-GFP fluorescence drops in boutons as synapsin I quickly disperses into neighboring axonal shafts and then slowly re-clusters (**Figures 7A–D**; Chi et al., 2001). Boutons treated with cPAF showed a much larger peak synapsin I dispersion (39.1 ± 1.6%; decaying with τ = 31.22 s) than vehicle treated boutons (31.3 ± 1.4%; decaying with τ = 23.14 s; **Figure 7D**). As synapsin I dispersion is calcium sensitive and regulated by phosphorylation of synapsin I (Chi et al., 2001, 2003; Cesca et al., 2010), we examined whether cPAF altered the levels of synapsin I phosphorylation at two sites known to be important for synapsin I binding to synaptic vesicles (Site1-ser9 and Site 3-Ser 603). Indeed, cPAF increased the level of pSynapsin I at site 1 and 3 in a time dependent manner (**Figures 7E,F**). This occurred even in the presence of APV and CNQX used to block recurrent excitatory glutamatergic transmission through NMDA and AMPA receptors, respectively. Thus cPAF’s increase in presynaptic vesicle release is associated with enhanced synapsin I dispersion and phosphorylation.

## Discussion

In this work we specifically focused on PAF’s modulation of synaptic vesicle release. Our study used optical readouts of synaptic vesicle recycling and presynaptic calcium levels coupled with inhibition of postsynaptic glutamate receptors. This allowed us to isolate PAF’s effects within the presynaptic bouton without having to infer presynaptic activity from postsynaptic potentials or contend with the effects of recurrent activity. Our results show that PAF treatment enhances presynaptic vesicle exocytosis, raises calcium levels within the presynaptic bouton, and increases synapsin I phosphorylation and dispersion.

We showed that PAF’s presynaptic enhancement of neurotransmitter release was PKC dependent and that PAF increases the size and/or release kinetics of the RRP and the total recycling pools of synaptic vesicles. Other forms of presynaptic potentiation have also been shown to be PKC dependent. Specifically PKC has been implicated in increasing the size or refilling of the RRP and increasing the probability of release after high frequency stimulation (Korogod et al., 2007; Wierda et al., 2007; Fioravante et al., 2011; Chu et al., 2012, 2014; Cijsouw et al., 2014; Genc et al., 2014).

Our results show that PAF increases synapsin I dispersion upon stimulation and increases phosphorylation at sites 1 and 3. Synapsin I is phosphorylated by multiple kinases including CaMK I/II/IV, PKA, MAPK, Src, and Cdk1/5 and dephosphorylated by the phosphatases PP2A/B and calcineurin. These phosphorylation sites regulate trafficking of synaptic vesicles between the RRP and the recycling/resting pools (Cesca et al., 2010). Phosphorylation at sites 1 (PKA and CaMKI/IV) and 3 (CaMKII) is associated with decreased binding of synapsin I to synaptic vesicles and actin and increased neurotransmitter release (Cesca et al., 2010). Mutation of sites 1 and 3 results in decreased dispersion of GFP-Synapsin I (Chi et al., 2001, 2003) and prevents an increase in the RRP size after post-tetanic potentiation (Valente et al., 2012). Phosphorylation of site 1 by PKA counteracts presynaptic depression and increases the recovery rate suggesting quicker movement of vesicles from the recycling/resting pool into the RRP (Menegon et al., 2006). Thus, PAF’s presynaptic potentiation coupled to increased phosphorylation of synapsin I sites 1 and 3 is congruent with other forms of presynaptic potentiation. These other studies also suggest that the phosphorylation of synapsin I at sites 1 and 3 is an important step to generating PAF’s enhanced presynaptic vesicle release.

We further show that PAF activates silent presynaptic boutons resulting in more synapses being active for the same stimulus. Presynaptic silent synapses are reversible and occur in several neurotransmitter systems (Crawford and Mennerick, 2012). Many signaling pathways that increase or decrease neurotransmitter release or alter the priming or organization of synaptic vesicle pools change the amount of silent synapses (Moulder et al., 2006; Doussau et al., 2010; Kim and Ryan, 2010; Crawford et al., 2012; Ramirez-Franco et al., 2014). Currently the function of presynaptic silent synapses is unknown but they may add an extra level of control for Hebbian plasticity or a mechanism of protection against excitotoxicity (Crawford and Mennerick, 2012).

Finally, we show that PAF increases the number of synaptic vesicles that are released from individual boutons due to a specific stimulus. This increase in presynaptic strength is likely beneficial for PAF’s putative physiological role in enhancing LTP and learning and memory (Izquierdo et al., 1995; Aihara et al., 2000). However, in neuroinflammatory diseases such as HAND and multiple sclerosis, PAF produced from infiltrating peripheral immune cells and activated microglia could amplify the presynaptic activity of neighboring neurons to potentially harmful levels. Our previous studies have shown that in the presence of PAF, a physiological stimulus can cause excitotoxic damage to postsynaptic dendrites leading to spine loss and dendritic varicosities (Bellizzi et al., 2005). In addition to this structural damage, PAF can impair further synaptic plasticity as LTP induced by high frequency stimulation is occluded by chronic PAF treatment (Wieraszko et al., 1993; Bellizzi et al., 2005; Lu et al., 2007). The structural damage and impaired plasticity connected to chronically elevated PAF likely contribute to some cognitive impairment accompanying neuroinflammation. Pharmacological or genetic inhibition of the PAFR in rodent models of neuroinflammatory disease results in less neuronal injury and inflammation (Kihara et al., 2005; Farooqui et al., 2006; Belayev et al., 2008; Eggert et al., 2009; Musto and Samii, 2011; Rodrigues et al., 2011; Okubo et al., 2012; Motoyama et al., 2013) and better performance in a learning and memory task (Liu et al., 2007). Thus, PAFR antagonism may have positive disease-modifying outcomes in neuroinflammatory diseases. Our investigation of the intracellular mechanisms of PAF’s presynaptic enhancement provides novel insights into how PAF alters neuronal communication at the synapse that under pathological conditions may result in neuronal injury, impaired cognition, and the feed forward cycle of inflammation.

## Author Contributions

The study was designed by JH, SL, and HG. JH performed and analyzed experiments. JH, SL, and HG prepared the manuscript.

## Conflict of Interest Statement

The authors declare that the research was conducted in the absence of any commercial or financial relationships that could be construed as a potential conflict of interest.
