# Corrigendum: Platelet Activating Factor Enhances Synaptic Vesicle Exocytosis Via PKC, Elevated Intracellular Calcium, and Modulation of Synapsin 1 Dynamics and Phosphorylation

**DOI:** 10.3389/fncel.2016.00113

**Published:** 2016-05-04

**Authors:** Jennetta W. Hammond, Shao-Ming Lu, Harris A. Gelbard

**Affiliations:** Center for Neural Development and Disease, University of RochesterRochester, NY, USA

**Keywords:** platelet activating factor, PKC, synapsin, synaptic vesicles, calcium

In this article, one of our funding sources was reported incorrectly as P30 AI078494. The correct funding source is P30 AI078498. This correction does not affect the data or conclusions contained in the manuscript.

## Author contributions

All authors approve this corrigendum.

### Conflict of interest statement

The authors declare that the research was conducted in the absence of any commercial or financial relationships that could be construed as a potential conflict of interest.

