# Supplementary material for: Platelet Activating Factor Enhances Synaptic Vesicle Exocytosis Via PKC, Elevated Intracellular Calcium, and Modulation of Synapsin 1 Dynamics and Phosphorylation
Source: Front Cell Neurosci. 2016 Jan 8;9:505. doi: 10.3389/fncel.2015.00505 (PMC4705275; doi:10.3389/fncel.2015.00505)
Supplement: Supplementary file 1 [file Data_Sheet_1.DOCX]

Supplementary Material

Platelet activating factor enhances synaptic vesicle exocytosis via PKC, elevated intracellular calcium, and modulation of synapsin 1 dynamics and phosphorylation

Jennetta W. Hammond^*^, Shao-Ming Lu, Harris A. Gelbard

*** Correspondence:** jennetta_hammond@urmc.rochester.edu

# Supplementary Material and Methods

Primary hippocampal cultures were processed for immunofluorescence as outlined in primary article using these primary antibodies: PAFR (Bioss bs-1478R), MAP2 (Sigma M4403), GFAP (Neuromics CH22102), vGlut (EMD Millipore 5905), PAFR (Cayman Chemical 160602). When labeling surface PAFR, cells were incubated with the PAFR antibody (Cayman Chemical) for 18 hours before cells were permeabilized. Then cells were incubated with 0.1% triton-X for 5 minutes followed by co-staining with vGlut and then secondary antibodies. When PAFR blocking peptide was used, the PAFR antibody (Cayman Chemical) was incubated with PAFR blocking peptide (Cayman Chemical 160604) for 2 hours before incubating with cells.

**2 Supplementary Figures**

**Supplementary Figure 1.**  PAFR immunofluorescence in hippocampal neuronal cultures. A) PAFR is found in neurons and astrocytes: PAFR (green, antibody produced by Bioss); Map2 (blue, neuronal marker); GFAP (red, astrocyte marker). B) A second polyclonal PAFR antibody (produced by Cayman chemicals) that targets a separate PAFR epitope shows a similar staining pattern in neuronal cultures as the PAFR antibody from Bioss. Images show hippocampal neuronal cultures stained for vGlut (red; presynaptic marker) and surface PAFR (green; Cayman chemical). Images on far right are enlarged images of the boxed region shown in the merged image. C and D) Controls for PAFR immunofluorescence. C) Incubating the Cayman Chemical polyclonal PAFR antibody with a blocking peptide peptide before labeling neuronal cultures reduces the PAFR staining (green). Cells were co-stained with vGlut (red). D) Same as B except no PAFR antibody was added. This is a control for the background staining produced by the secondary antibody. (Scale bars in A-D = 10µm; except for far right images in B where scale bar = 2µm).
