# Supplementary figures and images for: Platelet Activating Factor Enhances Synaptic Vesicle Exocytosis Via PKC, Elevated Intracellular Calcium, and Modulation of Synapsin 1 Dynamics and Phosphorylation
Source: Front Cell Neurosci. 2016 Jan 8;9:505. doi: 10.3389/fncel.2015.00505 (PMC4705275; doi:10.3389/fncel.2015.00505)

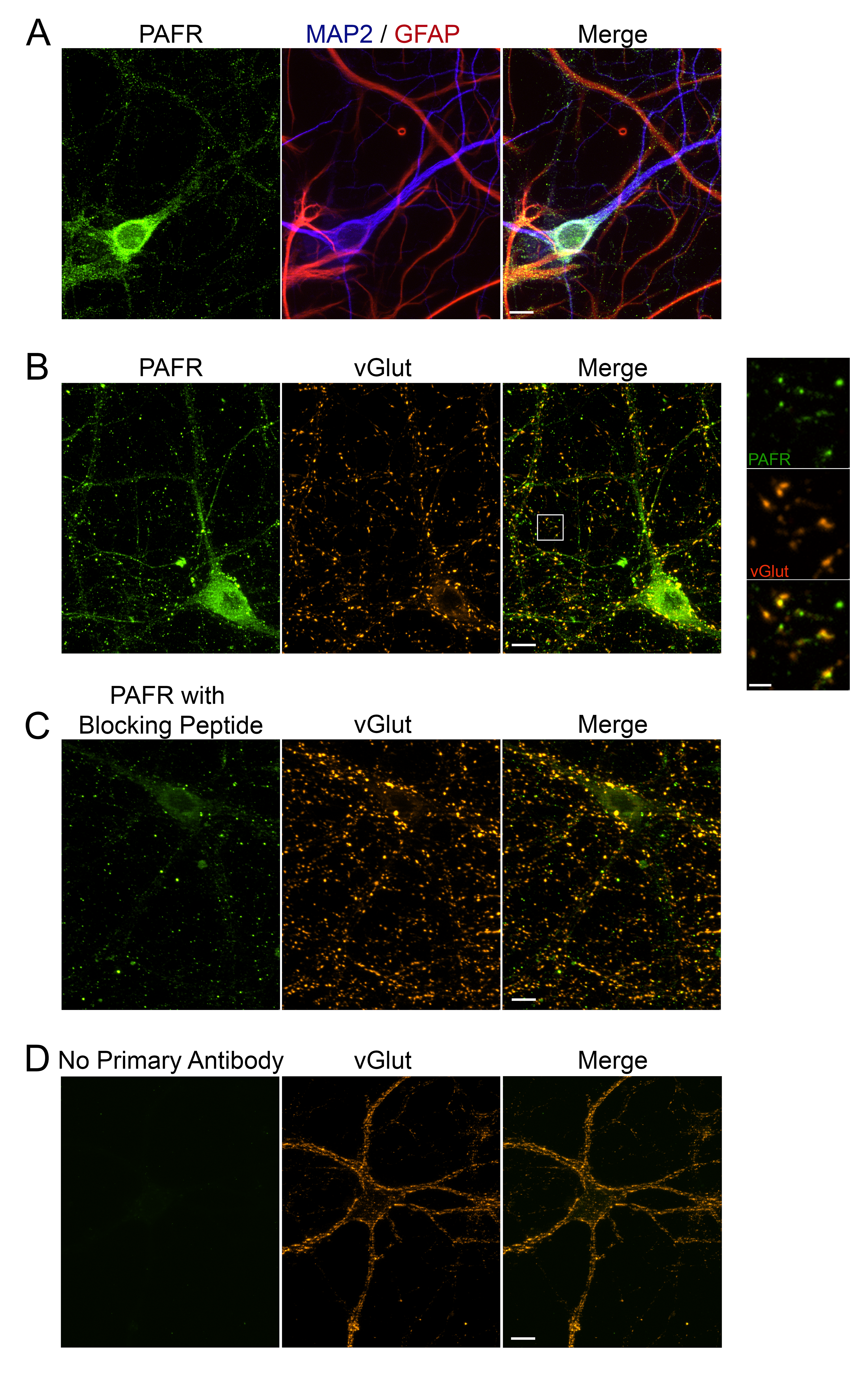

Supplement: Supplementary file 2 [file Supplementary_Figure_1.TIF]
